# Intrinsic immunity against retrotransposons by APOBEC cytidine deaminases

**DOI:** 10.3389/fmicb.2013.00028

**Published:** 2013-02-20

**Authors:** Atsushi Koito, Terumasa Ikeda

**Affiliations:** Department of Retrovirology and Self-Defense, Faculty of Life Sciences, Kumamoto UniversityKumamoto, Japan

**Keywords:** APOBEC1, APOBEC3, AID, retrovirus, HIV-1, LINE-1, retroelements, endogenous retrovirus

## Abstract

Over 40% of the human genome is recognizable as having been derived from ancient retroelements, transported by an intracellular copy-and-paste process involving an RNA intermediate, with an additional few percent classified as DNA transposable elements. Endogenous retroviruses are long terminal repeat (LTR)-type retroelements that account for ~8% of human genomic DNA. Non-LTR members are present at extremely high copy numbers, with ~17% of the human genome consisting of long interspersed nuclear elements (LINEs). These LINEs modify vertebrate genomes not only through insertions, but also by the indirect replication of non-autonomous retrotransposons, such as short interspersed nuclear elements. As expected, vertebrate intrinsic immunity has evolved to support a balance between retroelement insertions that confer beneficial genetic diversity and those that cause deleterious gene disruptions. The mammalian cytidine deaminases encoded by the *APOBEC3* genes can restrict a broad number of exogenous pathogens, such as exogenous retroviruses, and the mobility of endogenous retroelements. Furthermore, APOBEC1 from a variety of mammalian species, which mediates the cytidine (C) to uridine (U) deamination of apolipoprotein B (apoB) mRNA, a protein involved in lipid transport, also plays a role in controlling mobile elements. These mammalian apoB mRNA-editing, catalytic polypeptide (APOBEC) cytidine deaminases, which can bind to single-stranded DNA (ssDNA) as well as RNA, are able to insert mutations into ssDNA and/or RNA as a result of their ability to deaminate C to U. While these APOBEC cytidine deaminases with DNA mutagenic activity can be deleterious to cells, their biological modifications, such as protein–protein interactions and subcellular localization, in addition to their ability to bind to RNA, appear to have conferred a role for APOBECs as a cellular defense system against retroviruses and retroelements. In support of this notion, the expansion of the single *APOBEC3* gene in mice to the seven *APOBEC3* genes found in primates apparently correlates with the significant enhancement of the restriction of endogenous retroelements seen in primates, including humans. This review discusses the current understanding of the mechanism of action of APOBEC cytidine deaminases and attempts to summarize their roles in controlling retrotransposons.

## INTRODUCTION

The ability of members of the apolipoprotein B (apoB) mRNA-editing, catalytic polypeptide (APOBEC) family to confer intrinsic immunity against mobile elements was initially recognized for human APOBEC3G, which can block the replication of a human immunodeficiency virus type 1 (HIV-1) mutant lacking the virus infectivity factor (*vif*)**gene (Sheehy et al., 2002). APOBEC3 cytidine deaminases form one element of the cellular machinery that plays a role in the intrinsic restriction of two distinct classes of endogenous retroelements: non-long terminal repeat (non-LTR) retroelements, such as long interspersed nuclear elements (LINEs) and LTR retrotransposons (for reviews, see Holmes et al., 2007; Chiu and Greene, 2008; Koito and Ikeda, 2011, 2012). There is increasing evidence supporting the notion that the primary function of APOBEC3 cytidine deaminases could be to prevent the propagation of these intracellular mobile elements. Furthermore, APOBEC1 from non-human mammals, such as rodents and rabbits, has prominent intrinsic immune functions, regulating retroelements including HIV-1 in addition to its integral roles in editing its primary substrate, apoB mRNA (Bishop et al., 2004; Ikeda et al., 2008, 2011; Petit et al., 2009).

Increasingly detailed sequence analyses have revealed that a large portion of the mammalian genome is composed of non-LTR retrotransposons, with LINE-1 (L1), the most common LINEs, contributing to > 35% of the mass of the mammalian genomes (Lander et al., 2001; Waterston et al., 2002; Gibbs et al., 2004). Non-LTR retrotransposons, also called target-primed (TP) retrotransposons (Beauregard et al., 2008), predominantly undergo reverse transcription in the nucleus. These autonomous TP retrotransposons have modified host genomes not only by creating insertions, but also by their ability *in trans* to mediate the retrotransposition of cellular mRNAs to generate processed pseudogenes (copies of genes that are no longer functional) and short interspersed nuclear elements (SINEs). These SINE retrotransposons further constitute one of the main components of the genomic repetitive fractions.

On the other hand, the replication cycle of LTR retrotransposons, also called extrachromosomally-primed (EP) retrotransposons (Beauregard et al., 2008), is different, in which reverse transcription with the formation of virus-like particles (VLPs) occurs exclusively in the cytoplasm of infected cells. LTR retrotransposons, also called endogenous retroviruses (ERVs), which are structurally similar to HIV-1 and other infectious retroviruses, entered the germ line as infectious retroviruses at several time points during the evolution of many organisms. These mobile elements have been inherited through successive generations in the classical Mendelian manner and have been accumulated by reinfection and/or retrotransposition throughout evolution in the host genomes. This review summarizes and discusses the advances in the general knowledge of the APOBEC family proteins as a cellular defense mechanism against endogenous invaders of the genome.

## APOBEC FAMILY MEMBERS AS RESTRICTION FACTORS FOR NON-LTR RETROTRANSPOSONS

**FIGURE 1:**
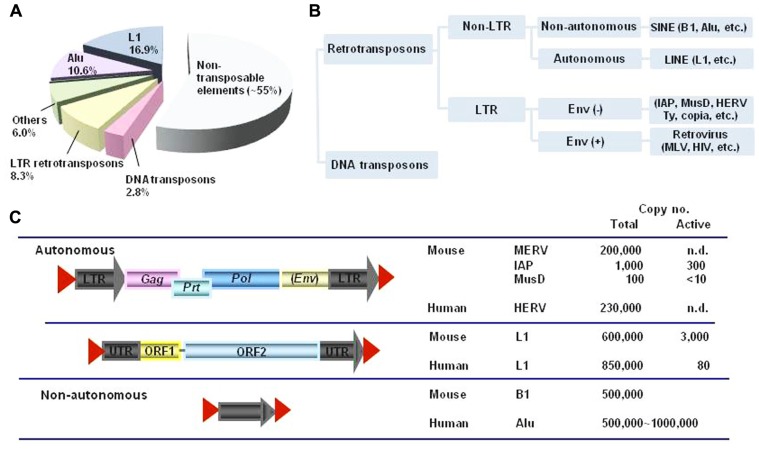
**The transposable element**. **(A)** The transposable element content of the human genome. Approximately 45% of the human genome is currently recognized as being derived from transposable elements. Transposable elements can be separated into two major classes: retrotransposons and DNA transposons (adapted with permission from Cordaux and Batzer, 2009). **(B)** Retrotransposons, which are found in both eukaryotes and prokaryotes, move into genomes via RNA intermediates with reverse transcriptase (RT). The majority of retrotransposons are non-LTR retrotransposons, such as short interspersed nuclear element (SINE), e.g., B1 and Alu elements, and long interspersed nuclear element (LINE), e.g., LINE-1 (L1). LTR retrotransposons, also called endogenous retroviruses (ERVs) are multicopy retroelements accounting for around 10% of the mammalian genome. Examples of LTR retrotransposons are human ERV (HERV), murine IAP, MusD, various Ty elements of *Saccharomyces cerevisiae* and copia of *Drosophila*. LTR retrotransposons usually lack a functional *env *gene, and are structurally similar to mammalian infectious retroviruses, such as MLV and HIV, which encode an envelope protein (Env) that facilitates their transmission from one cell to another. In contrast, ERVs either lack this gene or contain a remnant of an *env* gene, and can integrate into the genome at a new site within their cell of origin. **(C)** The structure of retroelements and their estimated occurrence in the murine and human genomes. ERVs contain slightly overlapping open reading frames (ORFs) for their group-specific antigen (Gag), protease (Prt), polymerase (Pol), and terminal LTRs. The *pol *genes encode a RT, ribonuclease H, and integrase to generate proviral cDNA from viral genomic RNA and to insert it into the host genome. L1 elements possess two ORFs. A ~6 kb functional L1 element contains an internal RNA polymerase II promoter in its 5′ untranslated region (UTR), followed by two ORFs. ORF1 encodes an RNA-binding protein (ORF1p) that is required for ribonucleoprotein particle (RNP) formation in the cytoplasm. ORF2 encodes a protein with endonuclease (EN) and RT activity (ORF2p). A short 3′-UTR is followed by a poly(A) tail, and the entire element is flanked by target site duplications (TSDs) indicated by red triangles. An Alu element is an example of a non-autonomous retrotransposon (adapted with permission from Esnault et al., 2005).

**FIGURE 2:**
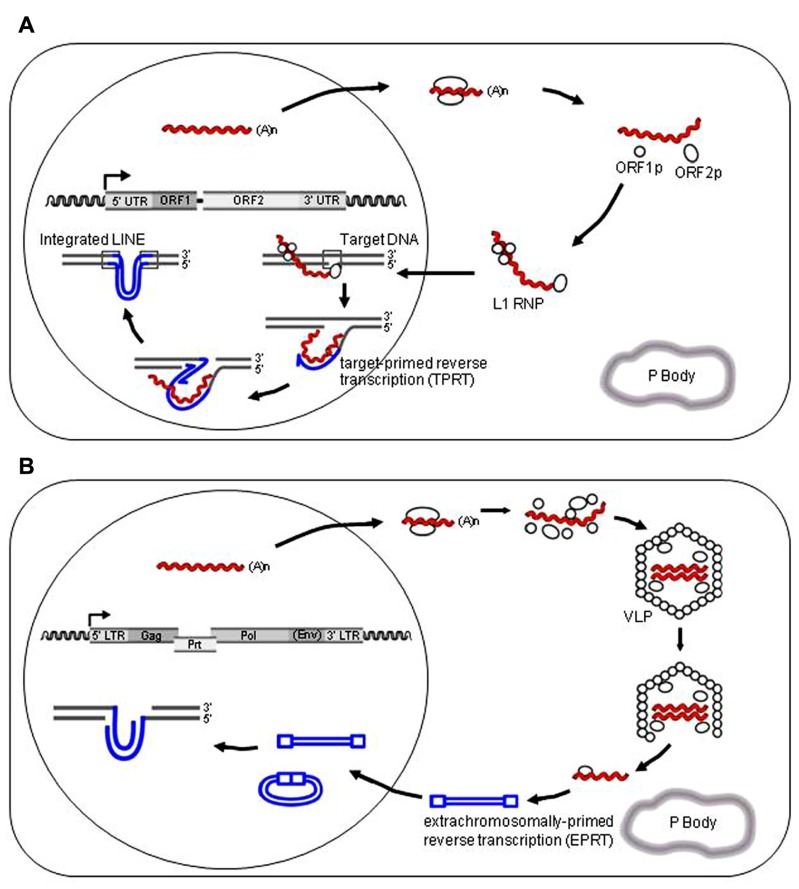
**Models for the retrotransposition cycle of retroelements**. **(A)** The retrotransposition pathway of L1 retroelements. A functional full-length L1 element contains an internal promoter in the 5′ untranslated region (5′ UTR) that initiates transcription. This is followed by two long open reading frames, ORF1 and ORF2, required for retrotransposition *in cis*. A short 3′ UTR is followed immediately by a poly(A) tail, and the entire element is typically flanked by target site duplications. ORF1p and ORF2p preferentially associate with their own encoding RNAs (“cis preference”) to form a RNP complex in the cytoplasm as a retrotransposition intermediate, and are critical for retrotransposition by a “copy and paste”**mechanism. L1 DNA synthesis in the nucleus is based on “target-primed reverse transcription (TPRT)” in which ORF2p nicks the target chromosomal DNA, and then uses the resultant 3′-OH to prime the reverse transcription of L1 RNA as a template. Human APOBEC3 proteins have been documented to associate with stress granules, Staufen granules, or P-bodies (gray enclosure); however, it appears that the inhibitory activity of human APOBEC3 proteins against L1 retrotransposition does not correlate with their P-body association.****L1 genomic RNA and reverse-transcribed DNA are indicated by red and blue colors, respectively. **(B)** The retrotransposition pathway of LTR-type retrotransposons (or endogenous retroviruses, ERVs), e.g., murine IAP, MusD, and yeast Ty1. The life cycle of these ERVs includes the formation of virus-like particles (VLPs) that remain intracellular. Reverse transcription of ERVs genomic RNA occurring in the cytoplasm is called “extrachromosomally-primed reverse transcription (EPRT)”, and is a complicated, multistep process. Reverse-transcribed single-stranded DNA is thought to be sensitive to APOBEC-mediated deamination activity. The proviral copies that have escaped degradation can integrate, but exhibit G-to-A mutations that render them defective for subsequent rounds of retrotransposition. P-bodies can influence the life cycle of ERVs and HIV-1 in either a positive or negative manner. ERVs genomic RNA and reverse-transcribed DNA are indicated by red and blue colors, respectively.

LINE-1 element is an autonomous retroelement, and comprises large fractions of the mammalian genomes (**Figure 1**). L1 is transcribed by RNA polymerase II to give a ~6-kb mRNA that encodes two open reading frames (ORF1p and ORF2p; Moran et al., 1996; **Figure 2**). ORF1p binds its own RNA to form a ribonucleoprotein (RNP) complex. In addition, ORF1p has a nucleic acid chaperon activity (Kolosha and Martin, 2003), which is also required for L1 retrotransposition (Martin et al., 2005). ORF2p has an endonuclease (EN) and reverse transcriptase (RT) domain, and forms a large RNP complex with the L1 RNA and ORF1p (Mathias et al., 1991; Feng et al., 1996; Kulpa and Moran, 2006). These structural alignments are well conserved in LINE-like elements from fish to mammals, although only mammals appear to limit L1 evolution to a single lineage (Furano et al., 2004). A comprehensive phylogenetic analysis based on the RT domain indicated that the LINEs can be divided into 11 distinct clades, and that the entire group was likely present at the beginning of the evolution of eukaryotes (Malik et al., 1999). An L1 homolog from lower eukaryotes**was demonstrated to be functional, indicating that L1s originated in the lower eukaryotes and expanded in many vertebrate species (Dong et al., 2009). These L1 retrotranspositions in various organisms have played, and continue to play, a significant role in shaping the host genomes through insertional mutagenesis, non-allelic recombination, and by mobilization *in trans* of non-L1 RNAs, such as SINEs (Bannert and Kurth, 2004; Kazazian, 2004; Cordaux and Batzer, 2009).

A family of host proteins that has been demonstrated to play a key role in the innate restriction of non-LTR retrotransposons is APOBEC. All members of human APOBEC3 family, APOBEC3A–APOBEC3H inhibit L1 to varying degrees (Kinomoto et al., 2007; Niewiadomska et al., 2007), with APOBEC3A and APOBEC3B being the most potent. Interestingly, the mechanisms underlying antiretroviral and anti-retrotransposon inhibition by the APOBEC family proteins appeared to differ, with the latter being independent of enzymatic activity. Similar DNA editing-independent anti-L1 activity had been reported for activation induced deaminase (AID) and APOBEC1 proteins in multiple mammalian species (MacDuff et al., 2009; Ikeda et al., 2011). The replication cycle of non-LTR retrotransposon L1 differs from that of LTR retroelements, with reverse transcription occurring within the cytoplasm that results in the formation of identifiable VLPs (**Figure 2**). To date, the exact step of the L1 replication cycle targeted by the APOBEC and other DNA editing-independent L1 restriction machineries has yet to be determined.

It has been documented that human APOBEC3G interacts with cellular RNAs; mRNAs, tRNAs, and rRNAs, and almost 100 different cellular RNA binding proteins to assemble into high-molecular-mass (HMM) RNP complexes that are converted to a low-molecular-mass (LMM) form by RNase treatment (for a review, see Chiu and Greene, 2008), although intracellular HMM complex formation does not appear to be a common feature among APOBEC family proteins. APOBEC3A was reported to localize in both the nucleus and the cytoplasm and to become associated with HMM complexes in the presence of L1 (Niewiadomska et al., 2007). APOBEC1 proteins were also found to exist in an HMM form in both the presence and absence of L1 (Ikeda et al., 2011). Notably, and in sharp contrast to APOBEC3G, the distribution of APOBEC1s was not affected by RNase treatment, suggesting that this single-domain cytidine deaminase may interact differently and/or more strongly with cellular RNAs.

Additionally, a homogenous cytoplasmic distribution of APOBEC3 proteins, along with discrete cytoplasmic foci referred to as mRNA-processing bodies (P-bodies), which involved in host mRNA degradation, translational repression, and microRNA-mediated RNA-silencing machinery has been demonstrated (Kozak et al., 2006; Wichroski et al., 2006; Gallois-Montbrun et al., 2007). The accumulation of APOBEC3 proteins in P-bodies can be explained by several possible mechanisms. The simplest possibility is that the concentration of APOBEC3 proteins reflects their binding to a subset of endogenous RNAs, which are translationally repressed and accumulate in P-bodies. For example, APOBEC3 proteins might interact with transcripts from endogenous retroelements, and the transcripts might be expected to be translationally repressed by miRNAs, thereby accumulating in P-bodies (Klattenhoff and Theurkauf, 2008). However, it appears that the inhibitory activity of APOBEC3 proteins against L1 retrotransposition does not correlate with the intracellular HMM formation or P-body association (Niewiadomska et al., 2007). Interactions between APOBEC3s and Ago1 and Ago2, proteins associated with the RNA interference pathway, were demonstrated (Gallois-Montbrun et al., 2007). Further, APOBEC3 proteins appeared to play a role in preventing the decay of miRNA-targeted mRNA from P-bodies, thus allowing for translation of these mRNA (Huang et al., 2007). These observations suggest that the recruitment of APOBEC family proteins into cellular sites of RNA metabolism and RNA-silencing pathways may represent one mechanism for regulating its activity as an inhibitor of retroelement mobility, and as a possible regulator of cellular RNA function. P-body associated host factor Moloney leukemia virus 10 (MOV10), an RNA helicase that belongs to the DExD box superfamily, is demonstrated to regulate L1 mobilization (Arjan-Odedra et al., 2012). In order to clarify the molecular mechanism through which L1 retrotransposition is inhibited, mainly in a deamination-independent manner, it is necessary to identify the exact step of L1 replication that is affected by these APOBEC proteins. Elucidation of this deamination-independent repressive activity of APOBECs on L1 retrotransposition may provide new insights into the consequences of deamination-independent HIV-1 inhibition by APOBEC3 proteins.

Despite the impact of L1 insertion on mammalian genome evolution, much of the L1 retrotransposition process, especially *in vivo*, remains unexplored. The majority of L1s are inactive due to the truncation, point mutations, and other rearrangements; however, it is estimated that the mouse and human genomes harbor 3,000 and ~100 copies of retrotransposition-competent L1 elements, respectively (**Figure 1**; Bannert and Kurth, 2004; Kazazian, 2004; Cordaux and Batzer, 2009). L1 retrotransposition has been demonstrated to result in the generation of novel polymorphisms in mammalian genomes, as well as a broad range of sporadic diseases in humans, including hemophilia A, Duchene muscular dystrophy, β-thalassemia, and colon cancer (Hancks and Kazazian, 2012). It was demonstrated that L1 RNA assembled into its RNP complex might be stable and could be carried over through fertilization using L1 transgenic rodent models, suggesting that the majority of *de novo* L1 retrotransposition usually occurs in early embryonic development (Kano et al., 2009). This scenario indicates that germ cells should have evolved several post-transcriptional defense mechanisms that strictly prevent the integration of transcribed L1 RNA into the genome. These defense mechanisms include post-transcriptional silencing via RNA interference (Yang and Kazazian, 2006), and APOBEC-mediated machinery may also contribute to the control of L1 retrotransposition in both early embryos and germ cells. In accord with this scenario, APOBEC3s mRNA is expressed in germ cells (Jarmuz et al., 2002; Mikl et al., 2005; Koning et al., 2009). Furthermore, APOBEC1 mRNA is expressed in germ cells in multiple mammalian species (Greeve et al., 1993; Ikeda et al., 2011), placing both APOBEC3 and APOBEC1 in a compartment where endogenous retroelements may have the greatest impact *in vivo*.

Short interspersed nuclear elements were also demonstrated to be sensitive to the restriction activity by human APOBEC3 family members (Bogerd et al., 2006a; Hulme et al., 2007; Tan et al., 2009). SINEs are transcribed by RNA polymerase III to give a ~300-nt non-coding RNA (Batzer and Deininger, 2002). Retrotransposition of non-autonomous retrotransposon SINEs depends on the L1 ORF2p with EN and RT activities (Dewannieux et al., 2003). It was reported that APOBEC3s do not require direct interactions with ORF1p with no known specific role in the L1 replication mechanism to inhibit the L1 retrotransposition (Lovsin and Peterlin, 2009). On the other hand, the interactions of ORF2p with APOBEC family proteins have not been addressed, thus far, since ORF2p within the cells is difficult to detect, even in the context of overexpression systems (Goodier et al., 2004). Therefore, the precise step(s) during which both LINE and SINE retrotransposition are affected by APOBEC family proteins are still unknown.

APOBEC1, as well as APOBEC3A and APOBEC3G, are able to inhibit nascent L1 DNA accumulation, suggesting that L1 reverse transcription, integration, and/or the intracellular movement of L1 RNPs are affected by these APOBEC enzymes (Kinomoto et al., 2007; Ikeda et al., 2011). The suppressive activity of these molecules against *de novo* L1 DNA synthesis occurred mainly in a deamination-independent manner, and was not affected by the subcellular localization of the proteins (Ikeda et al., 2011). It has not been described whether APOBECs interact with specific sequences in the Alu and L1 genomic RNA and/or L1-encoded ORF2p and/or host factor(s) that facilitate retrotransposition. These interactions might be able to interfere with the subsequent transport and/or nuclear import of cytoplasmic RNPs (for reviews, see Koito and Ikeda, 2011).

Although these genetic transposable elements have been mainly considered to be molecular fossils until recently, SINEs, which include murine B1 and human Alu elements, appear to play roles in the regulation of gene expression, the stress response, and proteome diversity (Chu et al., 1998; Ponicsan et al., 2010). This L1-mediated Alu retrotransposition has also been demonstrated to result in human diseases such as cancer (Dewannieux et al., 2003; Konkel and Batzer, 2010). Current studies are further emphasizing that SINEs insertions are involved in organizing and regulating intricate transcriptional pathways by dispersal of CCCTC-binding factor (CTCF), which acts as a master regulator of mammalian genomic boundaries that helps establish vertebrate insulators (Schmidt et al., 2012). Of note, increasingly detailed analyses of primate genomes informed that human genome contains threefold more abundant Alu sequences than that of the chimpanzee (Mikkelsen et al., 2005). Alu insertions appear to be particularly active in the human lineage after human–chimpanzee divergence, where they likely contribute to shaping some of the human-specific characteristics, such as brain size (Britten, 2010).

On the other hand, the L1 sequences of a transcript were demonstrated to possess a strong A-rich bias in the sense strand and serve as an evolutionary fine-tuner of the mammalian transcriptome by significantly decreasing RNA expression, and therefore protein expression (Han et al., 2004). Because L1 is also an abundant and broadly distributed mobile element, the inhibition of transcriptional elongation by L1 might profoundly affect the expression of endogenous human genes. Interestingly, recent studies further suggested that somatic genome mosaicism driven by L1 retrotransposition in the brain may influence brain activity (Baillie et al., 2011). The rapid expansion of non-LTR retrotransposons is likely to have had a major impact on the landscape and plasticity of the host genome, and significantly increased the rate of mammalian evolution, especially that of primates. Current hypotheses predict that the rapid expansion of L1 and Alu elements exerted strong positive selective pressure that resulted in the rapid evolution of APOBEC3s in primates around 30–50 million years ago (Jarmuz et al., 2002; Zhang and Webb, 2004).

## APOBEC FAMILY MEMBERS AS RESTRICTION FACTORS FOR LTR RETROTRANSPOSONS

Retroviruses that integrate into the germ line may be inherited vertically as ERVs, also known as LTR retrotransposons. Around 10% of the mammalian genomes is composed of these ERV elements (**Figure 1**), but the majority of them have been sufficiently degraded over time through mutations and deletions so that they are incapable of expressing infectious viruses (Bannert and Kurth, 2004; Kazazian, 2004; Jern and Coffin, 2008).

Following the discovery of the restriction activity of APOBEC3 proteins against HIV-1, similar DNA editing-dependent activities against murine ERVs, such as the intracisternal A particle (IAP) and MusD, were documented (Esnault et al., 2005, 2006; Bogerd et al., 2006b; Schumacher et al., 2008). The life cycle of IAP and MusD includes the formation of VLPs and reverse transcription in the cytoplasm of infected cells (**Figure 2**). IAP and MusD lack an extracellular phase and are not infectious, due to the absence of a functional *env* gene. The mouse genome contains numerous copies of IAP and MusD, of which about 300 IAP and 10 MusD copies are still active for autonomous intracellular retrotransposition (**Figure 1**; Dewannieux et al., 2004; Ribet et al., 2004). IAP and MusD mRNAs are expressed in germ cells, during early embryogenesis and in various tumor cells, and IAP VLPs are demonstrated to assemble and bud at the endoplasmic reticulum (ER) membrane (Heidmann and Heidmann, 1991; Dupressoir and Heidmann, 1996; Baust et al., 2003). In humans, IAP-like VLPs were detected in salivary tissues and in peripheral blood mononuclear cells, and appeared to be associated with Sjögren’s syndrome and CD4^+^ T cell deficiencies, respectively (Garry et al., 1990; Gupta et al., 1992). IAP insertions can lead to mutations and contribute to pathological processes. Therefore, it is critical for host cells to maintain their retrotransposition at low levels in order to maintain genome stability.

The inhibitory activity of APOBEC3 proteins against IAP and MusD appeared to be based, at least in part, on cytidine deamination. Consistent with reports indicating that the inhibitory activity of APOBEC3 proteins against exogenous retrovirus, such as HIV-1, was mediated by their selective incorporation into retroviral particles through an RNA-dependent interaction with the Gag protein, direct interactions between APOBEC3 proteins and IAP Gag have been demonstrated (Bogerd et al., 2006b). The molecular mechanisms responsible for the editing of reverse-transcribed DNA from endogenous and exogenous retroviruses appeared to overlap (Esnault et al., 2005; Bogerd et al., 2006b). Further, a genetic analysis demonstrated that some endogenous murine leukemia viruses (MuLVs) in the C57BL/6J genome bear the signatures of mutations induced by the murine APOBEC3 protein (Jern et al., 2007), indicating that these ERVs (MERVs) have been in conflict with APOBEC3 during murine evolution. The APOBEC3 enzyme was demonstrated to be dispensable for mouse development, survival and fertility (Mikl et al., 2005), although APOBEC3-knockout mice were more susceptible to Moloney MuLV (M-MuLV; Takeda et al., 2008; Low et al., 2009) and mouse mammary tumor virus (MMTV) replication (Okeoma et al., 2007). Although the murine APOBEC3 expressed in germ cells appears to be the likely mediator of the hypermutations observed in the MERVs, the participation of other cytidine deaminases in these modifications of the MERVs genome cannot be excluded at present. In addition, previous *ex vivo* studies on the effects of murine APOBEC3 on MuLV replication have been less clear (Doehle et al., 2005; Abudu et al., 2006; Zhang et al., 2008). MuLV is simple gammaretrovirus and does not encode any known *vif* analog. However, murine APOBEC3 does not induce obvious cytidine deamination when incorporated into MuLV virions. It is proposed that MuLV has evolved yet an unidentified mechanism for blocking the ability of APOBEC proteins to mediate deamination-dependent hypermutation (Browne and Littman, 2008, Rulli et al., 2008).

AID from multiple species, including lower vertebrates such as fish, and APOBEC1 proteins from several mammalian species were also found to possess the capacity to inhibit murine IAP and MusD elements (Esnault et al., 2006; MacDuff et al., 2009; Ikeda et al., 2011). These results raise the possibility that not only APOBEC3 proteins, but also AID and APOBEC1 cytidine deaminases participate in the intrinsic immunity of various vertebrates against the retrotransposition of endogenous and exogenous retroviruses. The catalytic activity of APOBEC1 appears to be critical for this repressive activity (Ikeda et al., 2008, 2011).

P-bodies appear to influence viral life cycles, including those of LTR retrotransposons within host cells, in either a positive or negative manner (reviewed in Beckham and Parker, 2008), although the underlying mechanism is not fully understood. Indeed, P-bodies play a major role during the replicative cycles of LTR retrotransposon Ty elements in yeast (Beliakova-Bethell et al., 2006). P-body is subcellular foci where Ty mRNA and proteins aggregate to facilitate their assembly and replication. On the other hand, the siRNA-mediated knockdown of RNA-induced silencing complex (RISC) and P-body-associated proteins was demonstrated to increase HIV-1 replication and IAP retrotransposition (Chable-Bessia et al., 2009; Nathans et al., 2009;Lu et al., 2011). P-body-associated host factor MOV10 is also demonstrated to inhibit IAP retrotransposition (Lu et al., 2012).

It was suggested that HIV-1 preferentially packages newly synthesized human APOBEC3G, rather than the RNA-bound APOBEC3G found in P-bodies or in the HMM complex (Soros et al., 2007; Ma et al., 2011). The efficiency of packaging into HIV-1 particles appears to correlate with the ability of APOBEC3G to binds to HIV-1 Gag nucleocapsid (NC) domain and to require bridging to heterologous single-stranded RNAs such as Pol II-transcribed poly(A)^+^ RNA and several Pol III-transcribed RNAs (Bogerd and Cullen, 2008). Among Pol III-transcribed short, non-coding RNAs, human 7SL RNA and Y RNAs were demonstrated to promote HIV-1 Gag NC binding by APOBEC3G, while some highly structured RNA molecules, such as the tRNA and rRNA, failed to rescue APOBEC3G:NC complex formation. This RNA bridging by APOBEC3G, not RNA binding by NC, appears to render APOBEC3G competent to associate with HIV-1 NC.

So far, no active ERVs have been isolated in the human genome (HERVs), despite evidence for recent (<200,000 years) amplification (Bannert and Kurth, 2004). However, although none of the HERVs are replication-competent due to their accumulation of mutations (deletions, termination, and frame shifts), more than 20 independent HERV families, which include proviruses that belong to beta-, gamma-, and spuma-retrovirus families, have been identified (Tristem, 2000). HERV families have been classified by the tRNA specificity of their primer binding site (PBS; Blomberg et al., 2009). Many HERV families have lost the ability to transfer, however, several HERV elements, e.g., HERV-K, HERV-H, HERV-W, and HERV-L, possess intact ORFs that encode structural genes and retain the capacity to be translated under certain conditions, including embryonic development and disease states (Kurth and Bannert, 2010).

The relationship between HERV elements and human diseases has been widely discussed following the detection of various HERV genome-derived mRNA, proteins, and even viral particles in patients with several diseases (Nelson et al., 1999; Mayer, 2001; Christensen, 2010).****It has also been demonstrated that****HERVs exhibit complex interactions with exogenous infectious viruses, such as HIV-1 and herpesviruses (Christensen, 2010). Of note, the most recently active HERVs, known as the HERV-K family with homology to MMTV (Mayer and Meese, 2005), which were reconstituted on the basis of ancient HERV-K sequences, could be restricted by APOBEC3 proteins in an *ex vivo *assay for their mobility (Lee and Bieniasz, 2007; Esnault et al., 2008; Lee et al., 2008). Moreover, the genetic analyses demonstrated that ancient HERV-K elements carry clear footprints of the deamination activity by human APOBEC3G, and to a lesser extent, APOBEC3F. The optimal sequence context of G-to-A mutations was consistent with human APOBEC3s-mediated editing (Armitage et al., 2008). This analysis provided the physiological relevance of the observed *ex vivo* assay. Primate APOBEC3s have been subjected to strong positive selection throughout primate evolution, and the rapid expansion of this gene family was suggested to occur in primates (Sawyer et al., 2004; Zhang and Webb, 2004).

It is still unclear whether human APOBEC3s have shaped the HIV-1 genome, because the results have been conflicting. Obviously, modern retroviruses such as HIV-1 were not a driving force that facilitated this rapid expansion of the *APOBEC3* locus on human chromosome 22q13 over millions of years of primate evolution, since HIV-1 has emerged and entered into the human population during the last 100 years (Korber et al., 2000; Keele et al., 2006; Worobey et al., 2008). In accord with this, it was currently demonstrated that the highly targeted motifs by human APOBEC3G and 3F (e.g., TGGG [the underlined G in the plus strand is deaminated to A]) have not been removed by selective pressure, suggesting the lack of an evolutionary footprint left by human APOBEC3s on the HIV-1 genome (Ebrahimi et al., 2011), although several studies have documented the possibility that evolutionary pressure from human APOBEC3s has shaped the HIV-1 genome (Yu et al., 2004; Armitage et al., 2008).

Based on these findings, it is reasonable to consider that the rapid evolution of APOBEC3s in primates can be attributed to the strong positive selective pressure from their targets, endogenous retrotransposons such as L1 and Alu elements, and that their evolution has been further promoted by repeated retroviral infection, including HERVs. The *APOBEC3* locus appears to have undergone major expansion during the evolutionary radiation of primates (LaRue et al., 2008). In primate lineage, humans, chimpanzees, and rhesus macaques share similar *APOBEC3 *locus architectures, with a seven-protein coding capacity of analogous domain organization (OhAinle et al., 2006; LaRue et al., 2008), indicating that rapid expansion of the *APOBEC3 *locus started before the separation of hominoids from Old world monkeys such as rhesus macaques over 50 million years ago. These APOBEC3s in rhesus macaques are demonstrated to be packaged into and restrict HIV-1 and neutralized by the SIV mac239 Vif (Hultquist et al., 2011). This rapid expansion of the *APOBEC3 *locus in primates may have caused a dramatic decline in the retrotransposon expansion activity in primates, since 35–50 million years ago (Lander et al., 2001). This scenario also raises the question why these unique rapid expansions of the *APOBEC3* locus have occurred only in primate lineages, since “interspersed repeats” (copies of transposable retroelements) appear to be characteristically abundant in mammalian genomes****(Lander et al., 2001; Waterston et al., 2002; Gibbs et al., 2004). The activity of *APOBEC1* genes against retrotransposons may further expose evidence of a complex evolutionary history between APOBEC family and retrotransposons. The details of the expansion are not fully understood as the orthologs of many *APOBEC3 *genes have not been sequenced in other placental mammals. It is tempting to speculate that the function of APOBEC family proteins, such as APOBEC1, in intrinsic immunity has been taken over by expansion of APOBEC3s in primates, but they are conserved in the ancestor of placental mammals.

## CONCLUSION

The spectrum of biological functions of the APOBEC family is expanding. Several members of this family play important roles in intrinsic immunity by regulating the spread of foreign and endogenous nucleic acids through non-editing and editing mechanisms. In doing so, they balance the beneficial and deleterious effects of retrotransposition on the host genome. While the restriction activity of the APOBEC family against retroviruses and retroelements is a fairly recent discovery, earlier studies of the zinc-dependent deaminase superfamily of both prokaryotes and eukaryotes that act on nucleosides and nucleotides have provided evidence of a complex evolutionary history. These research findings on the ancient origins of the APOBEC family, and its presence in widely divergent vertebrate lineages provide further insights into the co-evolution of the APOBEC family and retrotransposons.

## Conflict of Interest Statement

The authors declare that the research was conducted in the absence of any commercial or financial relationships that could be construed as a potential conflict of interest.
